# Developing combinatorial multi-component therapies (CMCT) of drugs that are more specific and have fewer side effects than traditional one drug therapies

**DOI:** 10.1186/1753-4631-1-11

**Published:** 2007-08-30

**Authors:** Larry S Liebovitch, Nicholas Tsinoremas, Abhijit Pandya

**Affiliations:** 1 Florida Atlantic University, Center for Complex Systems and Brain Sciences, Center for Molecular Biology and Biotechnology, Department of Psychology, Department of Biomedical Science, Boca Raton, FL 33431, USA; 2 The Scripps Research Institute, Scripps Florida, Informatics, Jupiter FL 33458, USA; 3 Florida Atlantic University, Department of Computer Science and Engineering, Boca Raton, FL 33431, USA

## Abstract

Drugs designed for a specific target are always found to have multiple effects. Rather than hope that one bullet can be designed to hit only one target, nonlinear interactions across genomic and proteomic networks could be used to design Combinatorial Multi-Component Therapies (CMCT) that are more targeted with fewer side effects. We show here how computational approaches can be used to predict which combinations of drugs would produce the best effects. Using a nonlinear model of how the output effect depends on multiple input drugs, we show that an artificial neural network can accurately predict the effect of all 2^15 ^= 32,768 combinations of drug inputs using only the limited data of the output effect of the drugs presented one-at-a-time and pairs-at-a-time.

## Background

One mission of Nonlinear Biomedical Physics is to publish "suggesting articles" that illustrate the application of new nonlinear paradigms to the solution of problems in biology and medicine. Our goal in this "suggesting article" is to suggest a new way to think about medical therapies and drug discovery to cure human diseases.

Our long experience with linear systems has led us to believe that one well defined cause can be associated with a single, well defined, identifiable effect. Around 1900, Paul Ehrlich applied this approach to medicine. He coined the phrase "magic bullet" for his search for the cure of syphilis to mean a chemical that would attack only the syphilis bacteria and spare the host tissue completely [1,2]. Over these last hundred years medicine has focused on finding the single therapeutic intervention that would be the most efficacious with the fewest side effects.

But we know that biology is not linear, it is a network of highly nonlinear genomic and proteomic interactions. Some genes express protein transcription factors that bind to other genes (or other transcription factors) that regulate the expression of other genes. Proteins serve as reactants or products in complex networks of biochemical reactions or as enzymes catalyzing reactions. Everything is connected to everything else. In this beautiful and tangled complex web any therapeutic interaction spreads throughout the entire network of interactions. There is no single effect that can be associated with a single cause. A single therapeutic intervention does not produce a single desired effect, it produces many "side effects."

Rather than struggling against the complex biological network of interactions and trying to find magic bullets that hit only their selected targets and nothing else, we propose here to use the wonderful tangled complexity of the biological network to our advantage. By understanding the biological network of interactions, or more practically by determining enough of its properties from limited experimental data, we should be able to predict which multiple inputs into the network will interact with each other, in just the right way, to produce the specific effects that we seek to achieve with the fewest unwanted side effects. We call this new approach Combinatorial Multi-Component Therapies (CMCT). In this paper we illustrate this approach using a well-defined mathematical nonlinear model of a biological network to show how computational approaches can be used to analyze relatively limited information about the effects of drugs presented one-at-a-time and pairs-at-a-time, to determine the combinations of these drugs that will have the most desired effects.

## Motivation

The specific project described here was motivated by two applications. The first application involved the use of RNA interference (RNAi) technology, where the injection of double stranded RNA can silence the expression of genes with complementary sequences [3-5]. Thus, specific RNAi could in principle, be used to turn off the expression of specific targeted genes to produce specific therapeutic effects. But many genes express protein transcription factors (or co-transcription factors that work together with other transcription factors) that bind to the regulatory regions of genes which increase or decrease the expression of those genes. Thus, there is a complex Transcription Regulatory Network (TRN). The effect of changing the expression of any one gene will likely cascade throughout the TRN effecting the expression of many other genes. Thus, each RNAi targeted to silence a specific gene could effect many different genes and produce many different effects on the phenotype of a cell. How can this tangled web of interactions be understood and even used to our advantage to develop novel therapies? It was proposed to measure how the phenotype of a cell depends on the RNAi of many different genes presented one-at-a-time and in pairs-at-a-time. Comparing the dependency of the phenotype measure determined from a linear model where the RNAi was presented one-at-a-time to the actual results of the RNAi presented in pairs-at-a-time, would give important information on the nonlinear interaction of the genes across the TRN. In the spirit of the CMCT approach presented here, we asked whether we could use the limited information about the effects of the RNAi presented one-at-a-time and pairs-at-time to predict what combination of all RNAi's would maximize or minimize the phenotypical effect.

The second application involved the use of combinations of drugs in chemotherapy to treat cancers. Patients with similar types of tumors, as identified by biomarkers, are given different combinations of therapeutic agents. In the spirit of the CMCT approach presented here, we asked whether we could combine this heterogeneous information from the effects of different combinations of drugs in different patients to design the best mix of agents to treat different types and stages of cancers.

## Approach

Our goal in this paper is to test the concept that computational approaches using relatively limited data on the effects of inputs into a model nonlinear biological network can be used to accurately predict what combinations of inputs would produce a maximal or minimal effect. A successful test of this concept would then open the door to using similar computational approaches on actual experimental or clinical data to develop actual Combinatorial Multi-Component Therapies.

We constructed a number of different linear and nonlinear mathematical models of how the effective outputs of a biological network depend on the input drugs into the network. We then used those models to generate "data" of the output response to inputs presented one-at-a-time and pairs-at-a-time. From this limited data set, we then used a computational approach to predict what outputs we would expect for all combinations of inputs. We then compared those predictions with the exact result from the model. We found that for a (reasonably) realistic model of how the outputs depend on the inputs, our computational approach could accurately predict the outputs expected of all combinations of inputs.

## Methods

We tested a number of different mathematical models of how the output effects, f_i_, of a biological network depend on the drugs, di, presented as the inputs into the network. These included a linear model,

f_i _= a_i1_d_1 _+ a_i2_d_2 _+ a_i3_d_3 _ + ...

two nonlinear models that could be transformed into linear models by a logarithmic transformation,

fi=e[ai1d1+ai2d2+ai3d3+...]
 MathType@MTEF@5@5@+=feaafiart1ev1aaatCvAUfKttLearuWrP9MDH5MBPbIqV92AaeXatLxBI9gBaebbnrfifHhDYfgasaacH8akY=wiFfYdH8Gipec8Eeeu0xXdbba9frFj0=OqFfea0dXdd9vqai=hGuQ8kuc9pgc9s8qqaq=dirpe0xb9q8qiLsFr0=vr0=vr0dc8meaabaqaciaacaGaaeqabaqabeGadaaakeaacqqGMbGzdaWgaaWcbaGaeeyAaKgabeaakiabg2da9iabbwgaLnaaCaaaleqabaGaei4waSLaeeyyae2aaSbaaWqaaiabbMgaPjabigdaXaqabaWccqqGKbazdaWgaaadbaGaeGymaedabeaaliabgUcaRiabbggaHnaaBaaameaacqqGPbqAcqaIYaGmaeqaaSGaeeizaq2aaSbaaWqaaiabikdaYaqabaWccqGHRaWkcqqGHbqydaWgaaadbaGaeeyAaKMaeG4mamdabeaaliabbsgaKnaaBaaameaacqaIZaWmaeqaaSGaey4kaSIaeiOla4IaeiOla4IaeiOla4Iaeiyxa0faaaaa@4CB3@

fi=[d1]ai1[d2]ai2[d3]ai3...
 MathType@MTEF@5@5@+=feaafiart1ev1aaatCvAUfKttLearuWrP9MDH5MBPbIqV92AaeXatLxBI9gBaebbnrfifHhDYfgasaacH8akY=wiFfYdH8Gipec8Eeeu0xXdbba9frFj0=OqFfea0dXdd9vqai=hGuQ8kuc9pgc9s8qqaq=dirpe0xb9q8qiLsFr0=vr0=vr0dc8meaabaqaciaacaGaaeqabaqabeGadaaakeaacqqGMbGzdaWgaaWcbaGaeeyAaKgabeaakiabg2da9iabcUfaBjabbsgaKnaaBaaaleaacqaIXaqmaeqaaOGaeiyxa01aaWbaaSqabeaacqqGHbqydaWgaaadbaGaeeyAaKMaeGymaedabeaaaaGccqGGBbWwcqqGKbazdaWgaaWcbaGaeGOmaidabeaakiabc2faDnaaCaaaleqabaGaeeyyae2aaSbaaWqaaiabbMgaPjabikdaYaqabaaaaOGaei4waSLaeeizaq2aaSbaaSqaaiabiodaZaqabaGccqGGDbqxdaahaaWcbeqaaiabbggaHnaaBaaameaacqqGPbqAcqaIZaWmaeqaaaaakiabc6caUiabc6caUiabc6caUaaa@4E0D@

and a nonlinear model that cannot be simply transformed into a linear model by a logarithmic transformation,

fi=[2ai11+e−bi1d1+1−ai1][2ai21+e−bi2d2+1−ai2][2ai31+e−bi3d3+1−ai3]...
 MathType@MTEF@5@5@+=feaafiart1ev1aaatCvAUfKttLearuWrP9MDH5MBPbIqV92AaeXatLxBI9gBaebbnrfifHhDYfgasaacH8akY=wiFfYdH8Gipec8Eeeu0xXdbba9frFj0=OqFfea0dXdd9vqai=hGuQ8kuc9pgc9s8qqaq=dirpe0xb9q8qiLsFr0=vr0=vr0dc8meaabaqaciaacaGaaeqabaqabeGadaaakeaacqqGMbGzdaWgaaWcbaGaeeyAaKgabeaakiabg2da9iabcUfaBnaalaaabaGaeGOmaiJaeeyyae2aaSbaaSqaaiabbMgaPjabigdaXaqabaaakeaacqaIXaqmcqGHRaWkcqqGLbqzdaahaaWcbeqaaiabgkHiTiabbkgaInaaBaaameaacqqGPbqAcqaIXaqmaeqaaSGaeeizaq2aaSbaaWqaaiabigdaXaqabaaaaaaakiabgUcaRiabigdaXiabgkHiTiabbggaHnaaBaaaleaacqqGPbqAcqaIXaqmaeqaaOGaeiyxa0Laei4waS1aaSaaaeaacqaIYaGmcqqGHbqydaWgaaWcbaGaeeyAaKMaeGOmaidabeaaaOqaaiabigdaXiabgUcaRiabbwgaLnaaCaaaleqabaGaeyOeI0IaeeOyai2aaSbaaWqaaiabbMgaPjabikdaYaqabaWccqqGKbazdaWgaaadbaGaeGOmaidabeaaaaaaaOGaey4kaSIaeGymaeJaeyOeI0Iaeeyyae2aaSbaaSqaaiabbMgaPjabikdaYaqabaGccqGGDbqxcqGGBbWwdaWcaaqaaiabikdaYiabbggaHnaaBaaaleaacqqGPbqAcqaIZaWmaeqaaaGcbaGaeGymaeJaey4kaSIaeeyzau2aaWbaaSqabeaacqGHsislcqqGIbGydaWgaaadbaGaeeyAaKMaeG4mamdabeaaliabbsgaKnaaBaaameaacqaIZaWmaeqaaaaaaaGccqGHRaWkcqaIXaqmcqGHsislcqqGHbqydaWgaaWcbaGaeeyAaKMaeG4mamdabeaakiabc2faDjabc6caUiabc6caUiabc6caUaaa@7C48@

**Figure 1:**
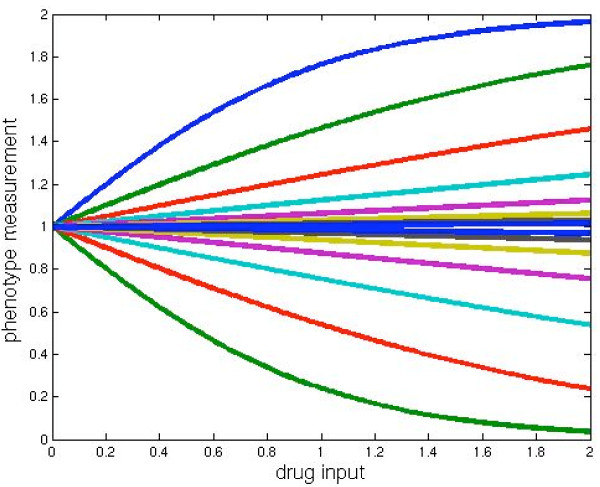
Each line in this graph shows how the output effect (vertical axis) of each term in the test model of eq. (4) depends on the drug input (horizontal axis). As can be seen, some drug inputs are excitatory (monotonically increasing) and others are inhibitory (monotonically decreasing). The combined output effect, f_i_, of all the input drugs is the product of all of these terms.

Each term in the model of eq. (4) is typical of an excitatory or inhibitory biochemical reaction that reaches a plateau as shown in Fig. 1. The output effect, f_i_, is the product of all these terms. For all these models, we determined how a limited amount of data, namely, the output effects, f_i_, computed by the inputs, d_i_, presented one-at-a-time and pairs-at-a-time, could be used to predict the outputs resulting from all combinations of inputs.

For the studies reported here we concentrate on the results that we obtained from the model in eq. (4) because it contains four important features typical of biological networks, namely: the simultaneous existence of both excitatory and inhibitory interactions, nonlinearities in the response of each node of the network, separation of scales, and nonlinear interactions between the nodes of the network. We now describe each of these four properties in more detail. 1) Networks of exclusively excitatory connections soon reach stable stationary states. For example, the dynamics of a network of nodes whose linear interactions are modeled by an adjacency matrix with only positive values converges to a fixed stable set of values of the nodes [6]. The complex dynamics in time of living systems are a result of mixed excitatory and inhibitory feedback which is found, for example, in the excitatory and inhibitory synapses between neurons in the nervous system, in the excitatory and inhibitory actions of transcription factors binding to DNA in gene expression, and in the excitatory and inhibitory actions of ligands binding to signal transduction proteins. In eq. (4) this is represented by including both positive and negative values of bij. 2) The effects of such electrical or chemical excitation and inhibition, are typically linear at low input levels, but then saturate at higher levels. The dependence of the output can be a highly nonlinear function of the input. The simplest way that such a nonlinearity can be incorporated is in the form of an exponential dependence which is done in eq. (4) through the exponential terms and their parameters bij. 3) Biological networks also display dynamics over a broad range of concentration, spatial, or temporal scales. For example, developmental cascades, gene expression, signal transduction, and biochemical protein modifications respectively take place over time scales of years, minutes, seconds, and milliseconds [7]. The range of concentration scales is represented in eq. (4) by choosing the parameters bij to be in a geometric progression so that the exponential dependence on the inputs covers a wide range of concentrations. 4) The effects induced by different genes and proteins are typically nonlinear rather than being simply additive. This is represented in eq. (4) by multiplying the effects of each term together, rather than adding them together. We cannot guarantee, and do not intend, this model to be a quantitate description of the RNAi or chemotherapy networks that motivated these studies. Rather, those two biological networks, and many others, are likely to have the same four general characteristics that are represented in the choice of the functional forms used in the model of eq. (4). Therefore, using this test model may give us results that would be representative of many different biological networks.

Given a limited set of data, many different computational approaches could be used to predict the output effects produced by different combinations of inputs. We choose to use an artificial neural network [8-11] because: 1) it can approximate any nonlinear input-output function (with a finite number of discontinuities) so that we do not need to assume any a priori functional form for the input-output relationship, and 2) there are well defined procedures to use the limited data to train the network (that is, to determine its parameters). Other computational approaches would be to fit the output effects produced by increasing numbers of inputs with a series of increasingly nonlinear functions, for example algebraic functions [12]. The advantage of the latter approach is that constraining the functional form makes it simpler and more reliable to use the limited data set to determine the parameters of that functional form. The disadvantage is that we may choose a functional form that is not a good match to the biological or model network. For this reason, namely, that we did not want to make any a priori assumptions about the input-output relationship, we chose to use the artificial neural network.

**Figure 2:**
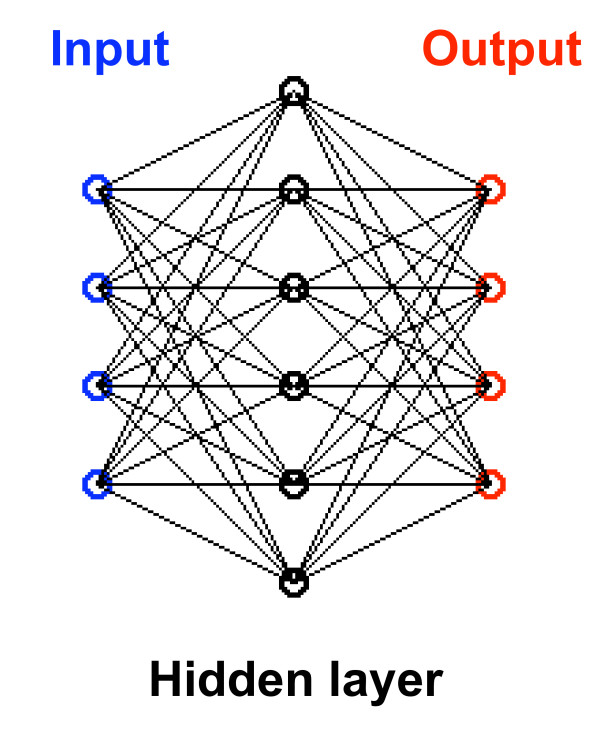
An artificial neural network was used to predict the output effects from all combinations of input drugs given limited information. The value of each drug is presented to one unit in the first layer of the network. The values of the units in each next layer are computed from the values of the units in the previous layer according to eq. (5) and eq. (6). The parameters of the network, the weights and biases, W_i, j_^N ^and B_i_^N^, are determined using the backpropagation algorithm to match the output effects from the input drugs presented one-at-a-time and pairs-at-a-time. Then the output effects are determined by presenting all possible combinations of drugs to the inputs. The network is represented here schematically. In the study most fully described in the text, there were 15 input units, 60 hidden layer units, and 1 output unit.

The artificial neural network that we used, is shown schematically in Fig. 2. It is a feed-forward three layer network consisting of input units in the first layer (N = 1), which are then connected to units in a "hidden layer" (N = 2), which are in tern connected to units in an output layer (N = 3). (The hidden layer gets its name from the fact that it does not receive inputs directly from the outside world nor does it provide outputs to the outside world.) The value of the j-th unit in layer N+1 is determined from the value of the units in the previous layer N by

XjN+1=f{∑i=1KWi,jNXiN+BiN}
 MathType@MTEF@5@5@+=feaafiart1ev1aaatCvAUfKttLearuWrP9MDH5MBPbIqV92AaeXatLxBI9gBaebbnrfifHhDYfgasaacH8akY=wiFfYdH8Gipec8Eeeu0xXdbba9frFj0=OqFfea0dXdd9vqai=hGuQ8kuc9pgc9s8qqaq=dirpe0xb9q8qiLsFr0=vr0=vr0dc8meaabaqaciaacaGaaeqabaqabeGadaaakeaacqqGybawdaqhaaWcbaGaeeOAaOgabaGaeeOta4Kaey4kaSIaeGymaedaaOGaeyypa0JaeeOzayMaei4EaS3aaabCaeaacqqGxbWvdaqhaaWcbaGaeeyAaKMaeeilaWIaeeOAaOgabaGaeeOta4eaaOGaeeiwaG1aa0baaSqaaiabbMgaPbqaaiabb6eaobaakiabgUcaRiabbkeacnaaDaaaleaacqqGPbqAaeaacqqGobGtaaGccqGG9bqFaSqaaiabbMgaPjabg2da9iabigdaXaqaaiabbUealbqdcqGHris5aaaa@4D1D@

where W_i, j_^N ^are the connection weights, B_i_^N ^are the biases, and the transfer function f is given by

f{Y}=tanh⁡(Yc)
 MathType@MTEF@5@5@+=feaafiart1ev1aaatCvAUfKttLearuWrP9MDH5MBPbIqV92AaeXatLxBI9gBaebbnrfifHhDYfgasaacH8akY=wiFfYdH8Gipec8Eeeu0xXdbba9frFj0=OqFfea0dXdd9vqai=hGuQ8kuc9pgc9s8qqaq=dirpe0xb9q8qiLsFr0=vr0=vr0dc8meaabaqaciaacaGaaeqabaqabeGadaaakeaacqqGMbGzcqGG7bWEcqqGzbqwcqGG9bqFcqGH9aqpcyGG0baDcqGGHbqycqGGUbGBcqGGObaAcqGGOaakdaWcaaqaaiabbMfazbqaaiabbogaJbaacqGGPaqkaaa@3CFE@

where c is a constant and typically c = 1. In principle, according to Kolmogorov's Theorem [8], such a network with n inputs, 2n +1 units in the hidden layer, and m units in the output layer can approximate any nonlinear input-output function of [0,1]^n ^--> R^m^. The algorithm of backpropagation [8,11] can be used to determine the parameter set of the weights and biases, W_i, j_^N ^and B_i_^N^, that best reproduce the limited amount of data in the training set, and hopefully will also make the best predictions of the output effects for new combinations of the inputs of the test set.

The artificial neural network was implemented in the Matlab Neural Network Toolbox [13] on a Macintosh Dual 2.5 GHz PowerPC G5 computer with MacOS 10.3.9. The test model for the dependence of the output effects on the input drugs was given by eq. (4) with aij = 1 and bij = 2, -2, 1, -1, 1/2, -1/2, ... . We used up to n = 15 units in the input layer, corresponding to up to 15 independent drugs. All the values of the inputs were either 0 or 1. The limited data set used to train the network consisted of the output effects computed from eq. (4) for all the inputs given one-at-a-time or pairs-at-a-time. The Bayesian regularization form of the backpropagation algorithm [14,15] implemented by trainbr in Matlab [13] was used to train the network, that is, to determine the parameters of the weights and biases, W_i, j_^N ^and B_i_^N^. Then those parameters were used to compete the output effects for all possible [0,1] combinations of the inputs and those results were compared to the values computed directly from the model in eq. (4). Four additional technical enhancements were used to improve the convergence of the backpropagation algorithm in training and the accuracy of the network in computing the output effects of new inputs in the test set: 1) the number of outputs was reduced to one, 2) the transfer function was softened from the input layer to the hidden layer by setting c = 4 in eq. (6), 3) the number of units in the hidden layer was increased to 4n, and 4) the network was trained and tested using the log_e_(outputs) which increased the accuracy by spreading out the output values that were near zero.

## Results

With the four additional enhancements described above, the network with 15 inputs, trained only on inputs presented one-at-a-time or pairs-at-a-time, accurately computed the output from all 2^15 ^= 32,768 combinations of [0,1] inputs with high accuracy, that is, 99% of all the input combinations were within 10% of the values of the output effects of the model of eq. (4).

## Summary

High throughput technologies are rapidly expanding our experimental capabilities to delineate the structure and dynamics of genomic and proteomic networks. With this understanding, and with sufficient computational power, we will be able to fully understand how single therapeutic inputs spread throughout these networks producing a cascade of effects. We will then be able to predict how multiple simultaneous therapeutic inputs into these networks will interact with each other, in just the right way, to produce specific targeted effects with reduced side effects. This will make possible a new paradigm of Combinatorial Multi-Component Therapies (CMCT) where we use the complex web of nonlinear biological interactions to our benefit, rather than fighting it in a frustrating search for illusory single magic bullets.

In this paper, we showed the output effects of 15 inputs presented one-at-a-time and pairs-at-a-time of a biologically reasonable model of a nonlinear network could be used to accurately compute the output effects of almost all the 2^15 ^= 32,768 possible combinations of inputs. This suggests that computational methods, perhaps suitably scaled up, may well be able to compute the effects of the complex combinatorial multi-drug therapies based on relatively limited experimental data. That is, we may not even need to understand the full details of biological networks to implement a rudimentary, but useful, form of CMCT.
